# DNMT1 facilitates growth of breast cancer by inducing MEG3 hyper-methylation

**DOI:** 10.1186/s12935-022-02463-8

**Published:** 2022-02-02

**Authors:** Xiaotao Zhu, Lin Lv, Mingzheng Wang, Chen Fan, Xiaofeng Lu, Miaomiao Jin, Shuguang Li, Fan Wang

**Affiliations:** 1 Department of Thyroid Breast Surgery, Jinhua Municipal Central Hospital, No. 365 East Renmin Road, Jinhua, 321000 Zhejiang China; 2 Department of Breast Surgery, Women and Children Branch of Jinhua Municipal Central Hospital, Jinhua, 321000 China

**Keywords:** DNMT1, MEG3, Methylation, Breast cancer, Growth

## Abstract

**Background:**

To understand the effect of DNMT1-mediated MEG3 promoter methylation on breast cancer progression.

**Methods:**

Expression of DNMT1, MEG3 and miR-494-3p was assayed by qRT-PCR and western blot. Methylation-specific PCR was used to examine MEG3 promoter methylation level. ChIP, RNA binding protein immunoprecipitation assay and dual-luciferase reporter gene assay were applied to verify interaction between DNMT1 and MEG3, miR-494-3p and MEG3 and OTUD4. CCK-8, wound healing and Transwell assays were used to detect biological functions of breast cancer cells. Tumor growth was observed by tumor xenograft model.

**Results:**

DNMT1 and miR-494-3p were highly expressed while MEG3 and OTUD4 were lowly expressed in breast cancer cells. Knockdown of DNMT1 inhibited progression of breast cancer cells by enhance MEG3 expression through demethylation. MEG3 could downregulate miR-494-3p expression, and OTUD4 was a target of miR-494-3p. Upregulation of MEG3 and downregulation of miR-494-3p both inhibited malignant behavior of cells in vitro. In addition, high MEG3 expression restrained growth of breast cancer in vivo.

**Conclusion:**

Briefly, our results demonstrated that, DNMT1 induced methylation of MEG3 promoter, and played a key role in breast cancer growth throughmiR-494-3p/OTUD4 axis. These findings provide new insights into molecular therapeutic targets for breast cancer.

**Supplementary Information:**

The online version contains supplementary material available at 10.1186/s12935-022-02463-8.

## Background

Breast cancer has the highest incidence and remains the first cause of cancer death among women worldwide, leading to 522,000 deaths since 2008 [1]. Breast cancer is a diversified disease classified according to histology, immunopathology, mRNA expression profile and miRNA expression features, rather than a single gene disease [2]. With the deepening of research on molecular biology, it is believed that the development of breast cancer is implicated in aberrant oncogene and tumor repressor-related signaling pathways[3–6]. Hence, a complete understanding of development mechanism of breast cancer is of great significance for finding suitable molecular therapeutic targets.

DNA hypermethylation can distinguish cancer cells from normal cells, which causes insensitivity of cancer cells to signals indicting growth inhibition and evades programmed cell death by inhibiting tumor suppressor genes [7]. DNA hypermethylation participates in occurrence and cell survival of breast cancer, and its initiation mechanism is the abberrant expression of DNA methyltransferases (DNMTs), including DNMT1, DNMT3a and DNMT3b [8–10]. DNMT1 is an important methyltransferase that is abnormally high expressed in dividing cells and affects the development of cancer [11]. An example is that DNMT1-regulated lncRNA ADAMTS9-AS2 can be used as a possible biomarker for glioma [12]. The epigenetic inhibition of MEG3, a DNMT1-mediated long non-coding RNA, inhibits p53 pathway in glioma [13]. LncRNA MIR210HG binding to DNMT1 to upregulate CACNA2D2, thus promoting proliferation and invasion of non-small cell lung cancer [14]. However, molecular mechanism of DNMT1 regulating breast cancer still requires further study.

The downregulation of DNMT1 has been reported to suppress progression of breast cancer cells [15], and DNMT1 also can downregulate maternally expressed gene 3 (MEG3) expression through increasing methylation level of MEG3 in breast cancer [16]. MEG3 is an imprinted gene expressed according to the maternal originand encodes a lncRNA [17]. Many studies show that lncRNAs serve as a competiting endogenous RNA (ceRNA) sponging miRNA and regulating target mRNA [18, 19]. LncRNA MEG3 can be a promising biomarker for ovarian cancer diagnosis and treatment by modifying the epithelial-mesenchymal transsition of ovarian cancer cells through sponging miR-219a-5p and modulating EGFR [20]. It has been reported that lncRNA MEG3 targets miR-421 to inhibit cell epithelial-mesenchymal transition (EMT) in breast cancer [21]. However, whether lncRNA MEG3 affects BC growth through DNMT1 and the molecular mechanisms involved in this process remain unclear.

Herein, we measured DNMT1, MEG3, miR-494-3p and OTUD4 expression, verified the interaction between them, and then discussed the impact of them on biological function of breast cancer cells. In addition, we also studied the methylation status of MEG3 promoter after DNMT1 silencing and the role of MEG3 on breast cancer growth in vivo (Additional file 1: Figure S1). These findings may provide new strategies for breast cancer treatment.

## Methods

### Bioinformatics analysis

RAID database and starBase database were used to obtain downstream regulatory miRNAs of MEG3 and the potential targeted binding sites of lncRNA-miRNA. GSE70905 dataset of breast cancer was acquired from GEO database, including 45 normal samples and 45 tumor samples. Normal samples were set as control and differential expression analysis was conducted by using R language “limma” package. *p* value was corrected by using FDR method. Differentilly expressed genes (DEGs) were screened out (|logFC|>  1 and *p* value  < 0.05). The downstram targets of miR-494-3p were analyzed by TargetScan, mirDIP and starBase databases. Targeted binding sites of miRNA-mRNA were consultated on the TargetScan database.

### Cell incubation

Human breast epithelial cell line MCF10A (3111C0001CCC000406), human breast cancer cell lines MDA-MB-231 (3111C0001CCC000014), SUM 149(HTX2301) and human embryonic kidney cell line 293 T (3111C0001CCC000010) were selected for this study. MCF10A, MDA-MB-231 and 293-T cell lines were provided by the cell resource center of Institute of Basic Medical Sciences, Chinese Academy of Medical Sciences. SUM 149 cell lines were purchased from Otwobio Biotech(Guang Zhou) INC. MCF10A cell line was cultured in DMEM-F12 medium. 293-T cell lines were incubated in MEM-EBSS (MEM Eagles with Earle’s Balanced Salts) medium. MDA-MB-231 cell line was placed in L15 medium (Leibovitz Medium). SUM 149 cell line was incubated in 89% DMEM plus 1% double antibody. The mediums were all purchsed from Hyclone and contained 10% fetal bovine serum (FBS).

### Lentivirus vector construction

MEG3 cDNA was cloned into pcDNA4 vector, while the short hairpin RNAs (shRNAs) targeting DNMT1, MEG3 and OTUD4 were cloned into PLKO.1 vectors. pPAX2 and pVSVG along with target vectors were co-transfected into 293 T cells to construct lantivirual vectors. After 24 h and 48 h of transfection, supernatant was harvested and filtered through a 0.45-μm membrane. The viral supernatant was added to medium in a ratio of 1:3 for viral infection. After 24 h, stably transfected cell lines were selected using 2 μg/ml purinomycin. All vectors, mimics and inhibitors were purchased from GenePharma (Shanghai, China). The scramble shRNA and empty pcDNA4 vector were used as negative controls, respectively. Sequences for sh-DNMT1, sh-MEG3 and sh-OTUD4 were detailed in Additional file 1. According to the preliminary experiments, the shRNA with a better interference efficiency was selected and the results were presented in the Results section.

### Dual-luciferase reporter gene assay

The 3′-UTR of MEG3 or OTUD4 was ligated to psiCHECK2 vector that was fused with luciferase gene and had been digested with XhoI and NotI restriction enzymes. The QuikChange multi-site-directed Mutagenesis kit (Stratagene, LaJolla, CA) was used to mutate targeted sites of miR-494-3p on 3′-UTR. Luciferase activities were determined by dual-luciferase assay (Promega), and Renilla luciferase activity was used for normalization of Firefly luciferase activity.

### RNA binding protein immunoprecipitation (RIP) assay

Magna RIP RNA-Binding Protein Immunoprecipitation kit (Millipore, Burlington, MA) was used for RIP assay. MDA-MB-231 cells were lysed, and then cell extracts were cultured with protein magnetic beads and incubated with 2 μg of Ago2 antibody (ab186733, 1:30, Abcam, UK) or control IgG antibody (ab205718, 1:50, Abcam, UK) overnight at 4 ℃. The immunoprecipitated RNA was purified. MEG3, miR-494-3p and OTUD4 expression was detected by qRT-PCR.

### Chromatin immunoprecipitation (ChIP) assay

DNMT1 enrichment in MEG3 promoter region was analyzed using ChIP kit (Millipore, USA). When the MDA-MB-231 cells reached 70–80% in confluence, 1% formaldehyde was added and cells were fixed for 10 min. Later, the cross-linked products were randomly fragmented of appropriate size by 10 s of ultrasonication for 15 cycles with an interval of 10 s. After centrifugation at 13,000 rpm at 4 ℃, the collected supernatant was transferred into 3 tubes and cultured with positive control antibody RNA polymerase II, negative control antibody IgG of normal mice (Abcam, UK) and methylation transferase specific antibody DNMT1 (Abcam, UK) overnight at 4 ℃, respectively. Protein Agarose/Sepharose was applied to precipitate endogenous DNA–protein complexes, and the supernatant was adsorbed after a short centrifugation. The non-specific complexes were washed and de-crosslinked overnight at 65 ℃. Phenol/chloroform was used to extract and purify DNA fragments. qRT-PCR was used to test combination of DNMT1 and MEG3 promoter region. Primer sequences were detailed in Additional file 1.

### Methylation-specific PCR (MSP)

Genomic DNA was treated with sodium bisulfite and DNA methylation was tested by MSP using EZ DNA Methylation-Direct kit (Zymo Research). Two primer groups were used to amplify the promoter region of MEG3 containing multiple CpG sites, and the primer sequences were shown in Table 1. Conditions of PCR reaction were: pre-denaturation at 95 ℃ for 10 min, followed by 35 cycles of 95 ℃ for 45 s, 56 ℃ [methylated reaction (M)]/45 ℃ [unmethylated reaction (U)] for 45 s and 72 ℃ for 45 s, and finally extension at 72 ℃ for 10 min. The reaction products were treated with agarose gel electrophoresis and images were captured for further analysis.

**Table 1:** Primer sequences for qRT-PCR and MSP

Genes	Primer sequences
miR-494-3p	F: 5′-GAAACATACACGGGAAAC C-3′
	R: 5′-GTGCAGGGTCCGAGG T-3′
U6	F: 5′-CTCGCTTCG GCAGCACA-3′
	R: 5′-AACGCTTCACGAATTTGC GT-3′
DNMT1	F: 5′-CGGCTTCAGCACCTCATTTG-3′
	R: 5′-AGGTCGAGTCGGAATTGCTC-3′
MEG3	F: 5′-ATCATCCGTCCACCTCCTTGTCTTC-3′
	R: 5′-GTATGAGCATAGCAAAGGTCAGGGC-3′
MSP-MEG3 (methylation)	F: 5′-TATGAGTTGTAAGCGGTAGAGTTC-3′
	R: 5′-TACGAACTTAACGAAAAAAAAATCAT-3′
MSP-MEG3 (non-methylation)	F: 5′-GAATATGAGTTGTAAGTGGTAGAGTTT-3′
	R: 5′-TACAAACTTAACAAAAAAAAATCATACT-3′
OTUD4	F: 5′-TTCTGATGTGGATTACAGAGGGC-3′
	R: 5′-ACGCATGTTGTCTTACTCCTGA-3′
GAPDH	F: 5′-GAGTCAACGGATTTGGTCGT-3′
	R: 5′-TTGATTTTGGAGGGATCTCG-3′

### Western blot

RIPA lysis buffer (Takara Biotechnology, Dalian, China) was used to isolate total proteins from cells. Totally 20 μg of proteins were isolated by 12% sodium dodecyl sulfate–polyacrylamide gel electrophoresis and transferred onto polyvinylidene fluoride membranes (Millipore Corp., Billerica, MA, USA). After blocked in TBS buffer containing 5% skim milk (50 mmol/l NaCl, 10 mmol/l Tris, pH7.4), the membranes were washed with TBST three times for 5 min each time and incubated with primary antibodies at 4 ℃ overnight. Primary antibodies were DNMT1 (Abcam, UK), OTUD4 (Abcam, UK) and GAPDH Abcam, UK). Then, the membranes were incubated with horseradish peroxidase-conjugated secondary antibody (Santa Cruz Biotechnology) at room temperature for 1 h. At last, immunoreactive proteins were treated with enhanced chemiluminescence reagent (Amersham, Little Chalfont, UK) and protein bands were analyzed using Amersham Imager 600 system (GE Healthcare Life Sciences, Shanghai, China).

### qRT-PCR

Total RNA extraction from cells was performed with TRIzol reagent (Invitrogen). The OD260/280 value of each RNA sample was determined by an UV spectrometer. RNA concentration was calculated and samples were stored at − 80 ℃. cDNA of mRNA was obtained by reverse transcription kit (RR047A, Takara, Japan), while cDNA of miRNA was obtained by miRNA First Strand cDNA Synthesis kit (B532451-0020, Shanghai Sangon Biotech, China). The samples were loaded using the SYBR^®^ Premix Ex TaqTM II (Perfect Real Time) kit (DRR081, Takara, Japan) and subjected to qRT-PCR reaction on a real-time fluorescence quantitative PCR instrument (ABI 7500, ABI, Foster City, CA, USA). PCR amplification procedure was set as below: pre-denaturation at 95 ℃ for 30 s, with 40 cycles of 95 ℃ for 5 s and 60 ℃ for 34 s. Each sample treatment was repeated in triplicate. Primers were synthesized by Shanghai Sangon Biotech Company (Table 1). Relative expression of target genes was calculated by 2^−ΔΔCt^ method with GAPDH or U6 as internal reference.

### CCK-8

A total of 3 × 10^3^ MDA-MB-231 cells were inoculated into 96-well plates. After transfection on day 1, 2, 3 and 4, CCK-8 reagent (10 μl) was added to each well. The plates were placed at 37 ℃ for 2 h, and the absorbance was read at 450 nm using a microplate reader (Bio-Rad, San Diego, CA, USA).

### Wound healing assay

Cell motility was detected by wound-healing assay, as mentioned in a previous study [22]. In brief, 2 × 10^5^ MDA-MB-231 cells were inoculated on 6-well plates and cultured at 37 ℃ for 16 h. The monolayer was scraped and cells were cultured in a fresh medium without FBS for 24 h. At last, three different fields of each well were observed and photographed under an inverted microscope to measure the scratch width. Relative scratch width  = (0–24 h) scratch width/0 h scratch width.

### Transwell invasion assay

As mentioned previously, changes in cell invasion were analyzed through Transwell assay[23]. In this assay, 8.0-μm Millipore Transwell chambers containing Matrigel were used. Firstly, 1 × 10^5^ MDA-MB-231 cells were resuspended in 200 μl medium without FBS and then inoculated into upper chambers. Next, 500 μl medium with 10% FBS was added to the lower chambers. After 48 h of culture, un-invading cells were removed, and invading cells were stained with crystal violet. Finally, 5 random fields were observed under an inverted microscope to calculate cell number.

### Nude mice experiment [24]

Ten 6-week-old BALB/c female nude mice were bought from Beijing HFK bio-technology (Beijing, China). The mice were randomly divided into two groups with 5 in each group. Then, 5 × 10^6^ MDA-MB-231 cells with oe-NC or oe-MEG3 were resuspended in 100 μl PBS and sequentially injected into each mouse by tail vein injection. After 35 days, the mice were euthanized by CO_2_ inhalation. Tumor volume was assessed by caliper measurement and calculated as follow: V  =  D  ×  d^2^  × 0.5 (D, longer diameter; d, shorter diameter). This experiment was approved by the Animal Care and Use Committee of Jinhua Municipal Central Hospital.

### Statistical analysis

Data were analyzed by using GraphPad Prism 6.0 (Lajolla, CA), and each experiment was repeated 3 times. Results were exhibited as mean  ±  standard deviation. Comparison between two groups was analyzed by Student’s *t *test, and the comparison among multiple groups was analyzed by one-way ANOVA. Statistically significant difference was set as *p*  < 0.05.

## Results

### Downregulating DNMT1 inhibits growth of breast cancer cells by promoting MEG3

**Fig. 1:**
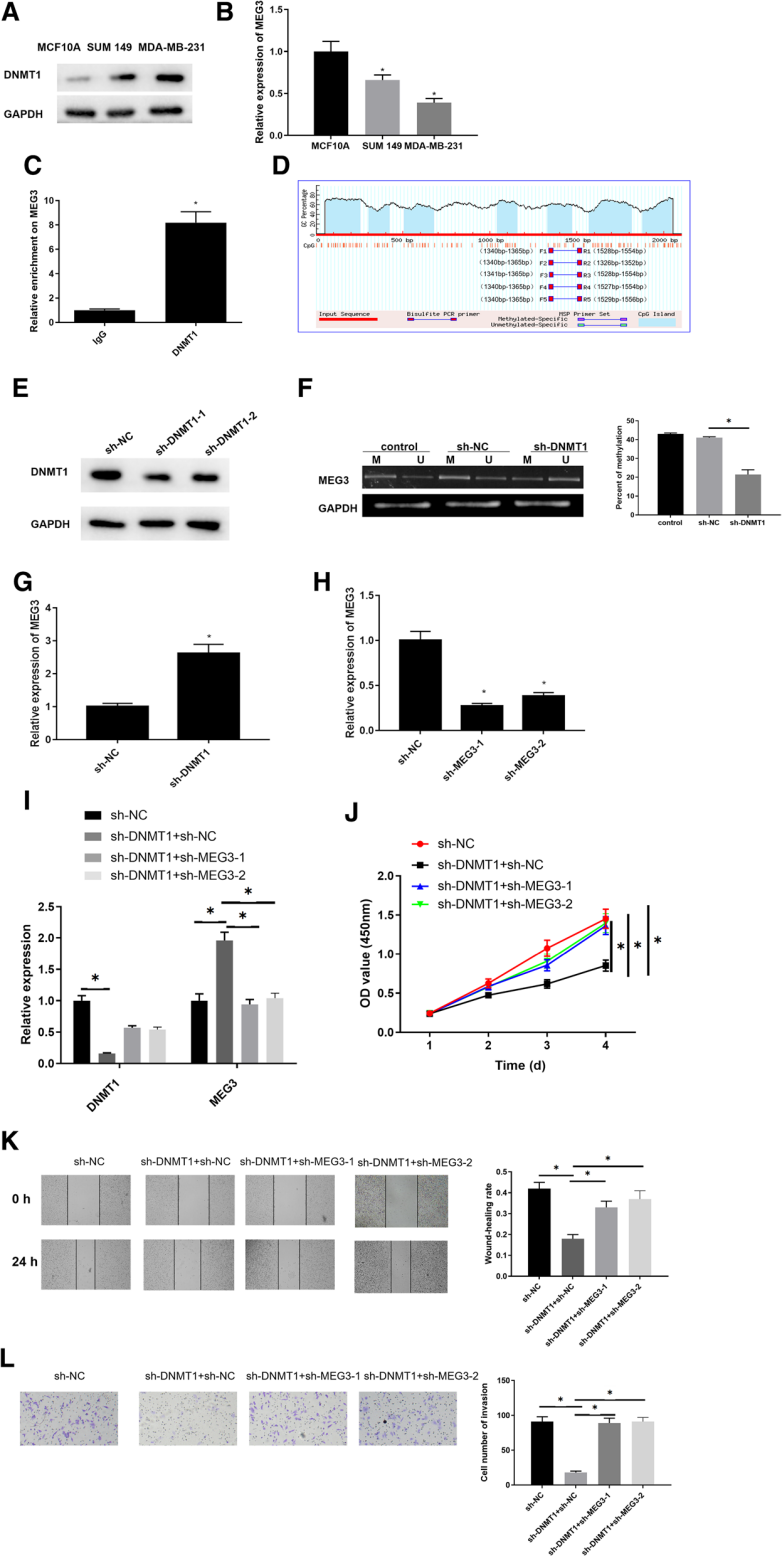
Downregulated DNMT1 inhibits progression of breast cancer cells through promoting MEG3. The **A** DNMT1 and **B** MEG3 expression levels in breast cancer cell lines (MDA-MB-231, SUM 149) and normal breast epithelial cell line MCF10A were detected by western blot and qRT-PCR; **C** ChIP assay was used to determine whether DNMT1 could bind to MEG3; **D** MEG3 methylation was determined by MethPrimer software; **E** Western blot was used to detect interference efficiency of the shRNAs targeting DNMT1; **F** MEG3 methylation level was tested by MSP (U, unmethylated reaction; M, methylated reaction; In control group, cells were not transfected with any plasmids); **G** MEG3 expression was detected after DNMT1 was silenced; **H** interference efficiency of the shRNAs targeting MEG3 was detected by qRT-PCR; **I** DNMT1 and MEG3 levels were detected by qRT-PCR in four groups (sh-NC, sh-DNMT1  +  sh-NC, sh-DNMT1  +  sh-MEG3-1, sh-DNMT1  +  sh-MEG3-2); **J** cell proliferation, **K** migration and **L** invasion were tested. *p  < 0.05

Current studies find that lncRNA MEG3 is regulated by DNMT1, and the promoter of MEG3 is methylated under the influence of DNMT1, while MEG3 appears to be hypermethylated and lowly expressed in tumors [16, 21, 25]. In view of this finding, we speculated that there may be a similar situation in breast cancer. Therefore, we further examined influence of the interaction of MEG3 with DNMT1 on the breast cancer cells. First, we utilized western blot (Fig. 1A) and qRT-PCR (Fig. 1B) to assess DNMT1 and MEG3 levels in breast cancer cell lines (MDA-MB-231, SUM 149) and normal breast epithelial cell line (MCF10A). It was showed that DNMT1 protein expression was significantly high in breast cancer cells, while MEG3 was significantly lowly expressed (*p*  < 0.05). MDA-MB-231 cell line with the lowest MEG3 expression was chosen for subsequent experiments. Afterwards, we assessed interaction of MEG3 and DNMT1 by ChIP (Fig. 1C). The results displayed that, compared with the IgG control group, DNMT1 enrichment in MEG3 promoter region was significantly increased (*p*  < 0.05). Besides, CpG islands were found in the MEG3 gene promoter region by analyzing 2100 bp nucleotide sequences near the MEG3 gene promoter region through the MethPrimer software, and the results indicated that MEG3 expression would be affected by promoter methylation (Fig. 1D). Then, DNMT1 interference efficiency in MDA-MB-231 cells was measured by western blot (Fig. 1E), and sh-DNMT1-1 was used in subsequent experiments for better interference efficiency. MEG3 methylation level detected by MSP (Fig. 1F) presented that MEG3 methylation level was significantly decreased upon DNMT1 knockdown. While qRT-PCR results revealed that MEG3 gene expression was remarkably up-regulated when silencing DNMT1 (Fig. 1G) (*p*  < 0.05). It indicated that silencing DNMT1 could promote MEG3 expression by inhibiting MEG3 promoter methylation.

It is reported that DNMT1 can promote the malignant progression of breast cancer [15], while MEG3 can elicit a suppressive effect [26]. In this study, we proved that DNMT1 could potentiate MEG3 promoter methylation in turn inhibiting MEG3 expression. Besides, aforementioned studies manifested that DNMT1 modulated breast cancer cell growth via repressing MEG3. To validate the speculation, the level of MEG3 expression was firstly interfered, and sh-MEG3-1 with a better interference efficiency as directed by qRT-PCR was selected for subsequent experiments (Fig. 1H). Then, expression levels of DNMT1 and MEG3 in four groups (sh-NC, sh-DNMT1  +  sh-NC, sh-DNMT1  +  sh-MEG3-1, sh-DNMT1  +  sh-MEG3-2) were detected by qRT-PCR (Fig. 1I). As demonstrated, DNMT1 expression was conspicuously down-regulated while the expression of MEG3 was remarkably up-regulated in the sh-DNMT1  +  sh-NC group relative to the sh-NC group. Besides, MEG3 was obviously down-regulated when DNMT1 and MEG3 were both silenced, and there was no significant diversity in DNMT1 expression, with a comparison of those in the sh-DNMT1  +  sh-NC group. Then, the results of CCK-8 (Fig. 1J), wound healing (Fig. 1K) and Transwell (Fig. 1L) assays displayed that silencing DNMT1 decreased cell activity, migratory and invasive abilities while these abilities were increased when DNMT1 and MEG3 were silenced simultaneously. In conclusion, silencing DNMT1 inhibited the malignant progression of breast cancer via up-regulating MEG3.

### miR-494-3p is targeted by MEG3 in breast cancer cells

**Fig. 2:**
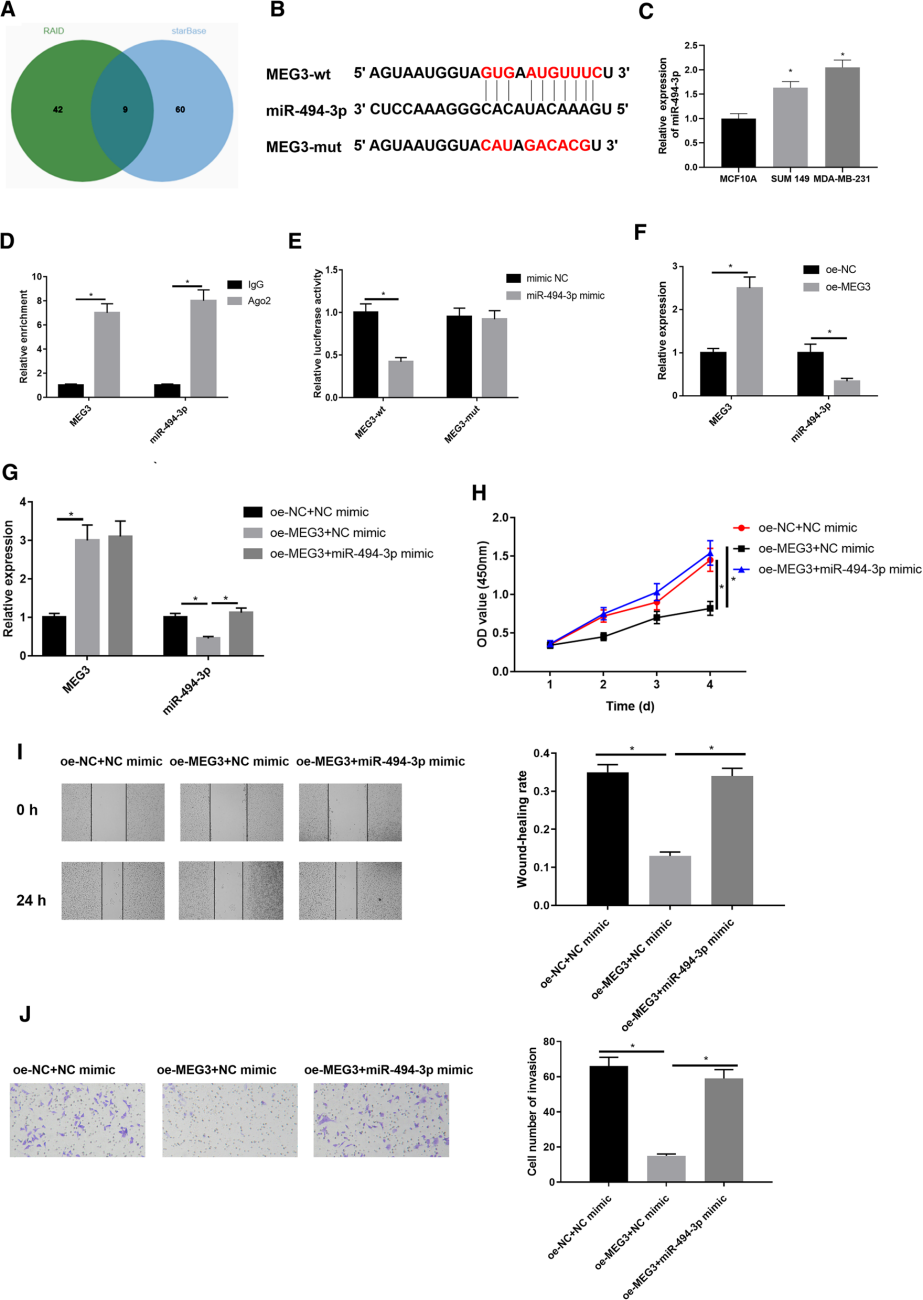
miR-494-3p is a target of MEG3 in breast cancer cells. **A** Potential downstream miRNAs of MEG3 along with the **B** binding sites of miR-494-3p on MEG3 were analyzed by bioinformatics; **C** miR-494-3p expression in breast cancer cell lines (MDA-MB-231, SUM 149) and normal breast epithelial cell line MCF10A was detected by qRT-PCR; The binding relationship of MEG3 with miR-494-3p was verified by **D** RIP and **E** dual-luciferase assays; **F** MEG3 and miR-494-3p levels in oe-NC group and oe-MEG3 group were detected by qRT-PCR; **G** MEG3 and miR-494-3p expression, **H** the cell proliferation, **I** migration and **J** invasion in oe-NC  +  NC mimic group, oe-MEG3  +  NC mimic group, and oe-MEG3  +  miR-494-3p mimic group were detected.*p  <  0.05

Studies have pointed out that MEG3 can play a regulatory role by acting as a ceRNA [27, 28]. To identify possible downstream modulatory molecules of MEG3, we predicted that miR-494-3p may be a possible target of MEG3 through bioinformatics analysis (Fig. 2A), and these two genes had binding sites (Fig. 2B). Moreover, a study put forward that miR-494-3p expression in tumors is notably increased [29]. Hence, miR-494-3p expression in breast cancer cell lines in vitro was further examined and found to be dramatically higher than that in control cell line (Fig. 2C). Afterward, we conducted RIP to validate the binding relationship between MEG3 and miR-494-3p. The result displayed that in comparison with the IgG group, quantity of MEG3 and miR-494-3p bound by Ago2 was significantly increased (Fig. 2D). Next, we conducted dual-luciferase assay to validate their binding relationship. The result illustrated that miR-494-3p overexpression markedly reduced luciferase activity of the MEG3-wt group but had no influence on that of the MEG3-mut group (Fig. 2E). Expression analysis was conducted by qRT-PCR for MEG3 and miR-494-3p in oe-NC group and oe-MEG3 group (Fig. 2F), demonstrating that miR-494-3p expression was remarkably down-regulated when MEG3 was upregulated, indicating that MEG3 targeted and negatively regulated miR-494-3p.

Then, rescue experiments were carried out to study modulation of MEG3/miR-494-3p axis in breast cancer cells. Firstly, MEG3 and miR-494-3p expression in 3 groups (oe-NC  +  NC mimic group, oe-MEG3  +  NC mimic group and oe-MEG3  +  miR-494-3p mimic group) was measured by qRT-PCR (Fig. 2G). Highly expressed MEG3 significantly suppressed miR-494-3p expression, but when MEG3 and miR-494-3p were overexpressed simultaneously, miR-494-3p expression was elevated greatly. Finally, a set of cellular experiments were done to assay the impact of MEG3/miR-494-3p axis on breast cancer cell viability, migration and proliferation. The results of CCK-8 (Fig. 2H), wound healing (Fig. 2I) and Transwell (Fig. 2J) assays displayed that MEG3 overexpression decreased cell activity, migratory and invasive abilities, while these abilities were recovered when MEG3 and miR-494-3p were overexpressed together (*p*  < 0.05).

### Silencing miR-494-3p inhibits growth of breast cancer cells by targeting OTUD4

**Fig. 3:**
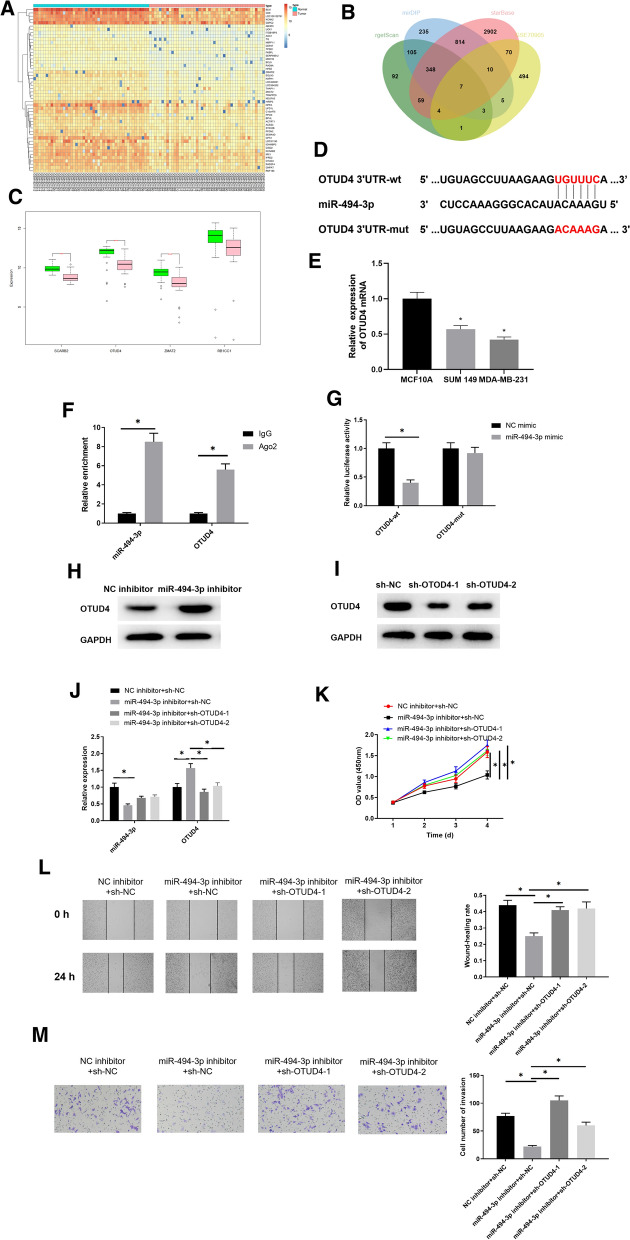
Silencing miR-494-3p restrains progression of breast cancer cells by targeting OTUD4. **A** DEGs in GSE70905 dataset from GEO database were analyzed; **B** Venn diagram of DEGs and predicted target genes of miR-494-3p; **C** differential expression of candidate genes in GSE70905 dataset; **D** the binding sites of miR-494-3p on OTUD4 3′UTR were analyzed by starBase database; **E** OTUD4 expression levels in breast cancer cell lines (MDA-MB-231,SUM 149) and normal breast epithelial cell line MCF10A were detected by qRT-PCR; **F** RIP and **G** dual luciferase assays were performed to verify targeting relationship between miR-494-3p and OTUD4; **H** OTUD4 protein expression in NC inhibitor and miR-494-3p inhibitor groups was detected by western blot; **I** Interference efficiency of sh-OTUD4 was tested by western blot; **J** miR-494-3p and OTUD4 expression levels, **K** the cell proliferation, **L** migration and **M** invasion in NC inhibitor  +  sh-NC, miR-494-3p inhibitor  +  sh-NC, and miR-494-3p inhibitor +  sh-OTUD4-1, and miR-494-3p inhibitor  +  sh-OTUD4-2 groups were measured. *p  < 0.05

To probe into downstream genes that may be affected by MEG3/miR-494-3p axis, we used bioinformatics methods to investigate downstream target mRNAs of miR-494-3p. Hence, DEGs in breast cancer dataset GSE70905 that was included in GEO database were analyzed (Fig. 3A), and downstream targets of miR-494-3p were predicted on bioinformatics databases. It was found that OTUD4 had specific binding sites of miR-494-3p and it was down-regulated in breast cancer (Fig. 3B–D). Similarly, in vitro cell experiments also indicated that OTUD4 mRNA in breast cancer cell lines was remarkably lower than that in normal cell line (Fig. 3E). We utilized RIP assay for verification of targeted relationship of miR-494-3p and OTUD4. The result in Fig. 3F revealed that compared with IgG group, quantity of miR-494-3p and OTUD4 bound by Ago2 was significantly increased. Dual-luciferase assay was conducted for further verification (Fig. 3G). The result displayed that luciferase activity of the OTUD4-wt group was notably decreased with miR-494-3p overexpression (*p*  < 0.05) but had no effect on that of the OTUD4-mut group (*p*  > 0.05). OTUD4 expression in NC inhibitor and miR-494-3p inhibitor groups was tested by western blot (Fig. 3H) and the results suggested that silencing miR-494-3p significantly up-regulated OTUD4 protein expression level (*p * < 0.05).

Rescue experiments were also used to test the regulatory effect of miR-494-3p/OTUD4 on breast cancer development. Firstly, sh-OTUD4-1, which had a better interference efficiency as judged by western blot, was chosen for further tests (Fig. 3I). miR-494-3p and OTUD4 expression in 4 groups (NC inhibitor  +  sh-NC group, miR-494-3p inhibitor  +  sh-NC group, miR-494-3p inhibitor  +  sh-OTUD4-1 group, and miR-494-3p inhibitor  +  sh-OTUD4-2 group) was detected by qRT-PCR (Fig. 3J). As displayed by result, silencing miR-494-3p significantly up-regulated OTUD4 expression, but when OTUD4 and miR-494-3p were silenced simultaneously, OTUD4 expression was decreased greatly (*p*  < 0.05). Finally, CCK-8, wound healing and Transwell assays were done for detection of influences of miR-494-3p/OTUD4 axis on breast cancer cell viability, migration, and invasion. As depicted in Fig. 3K–M, miR-494-3p knockdown decreased these cell properties, but the effects could be rescued by dual suppression of miR-494-3p and OTUD4 (*p*  < 0.05).

### MEG3 inversely modulates miR-494-3p to promote OTUD4 expression and inhibit growth of breast cancer cells

**Fig. 4:**
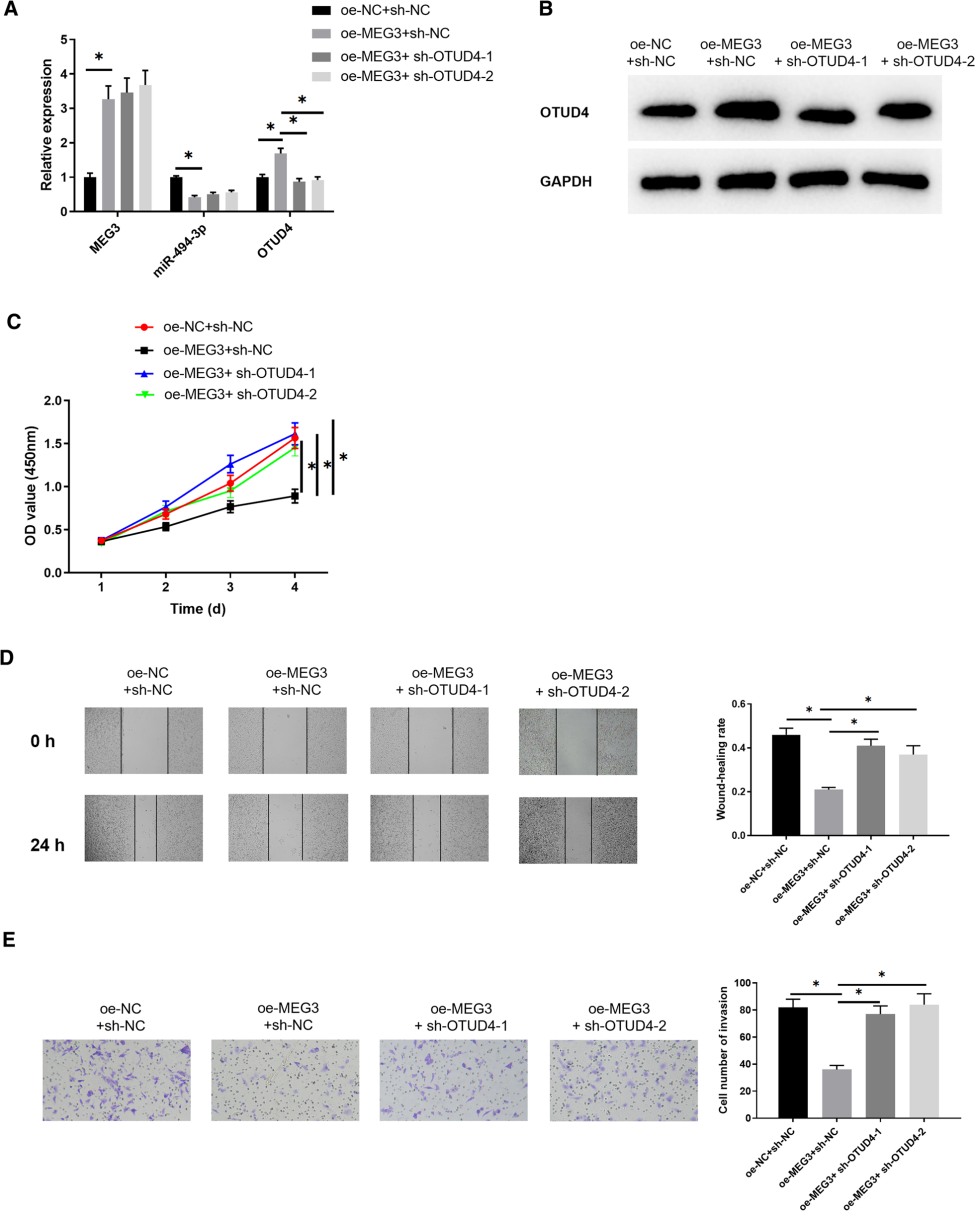
MEG3 inversely modulates miR-494-3p to promote OTUD4 expression and inhibits growth of breast cancer cells. **A** MEG3, miR-494-3p and OTUD4 expression in oe-NC  +  sh-NC, oe-MEG3  +  sh-NC, oe-MEG3  +  sh-OTUD4-1 and oe-MEG3  +  sh-OTUD4-2 groups was detected by qRT-PCR and **B** OTUD4 protein expression was tested by western blot; **C** cell proliferation, **D** migration and **E** invasion were measured. *p  < 0.05

For deeply understanding the influence of MEG3/miR-494-3p/OTUD4 as the regulatory axis on breast cancer cells, MEG3, miR-494-3p and OTUD4 expression in 4 groups (oe-NC  +  sh-NC group, oe-MEG3  +  sh-NC group oe-MEG3  +  sh-OTUD4-1 group and oe-MEG3  +  sh-OTUD4-2 group) was tested by qRT-PCR (Fig. 4A), and OTUD4 protein expression was measured by western blot (Fig. 4B). Overexpression of MEG3 significantly increased OTUD4 protein and mRNA expression levels, but reduced miR-494-3p expression. In comparison with oe-MEG3  +  sh-NC group, mRNA and protein expression levels of OTUD4 were greatly down-regulated in the oe-MEG3  +  sh-OTUD4 group, while MEG3 and miR-494-3p expression showed no significant difference (*p*  > 0.05). Finally, CCK-8, wound healing, and Transwell assays were completed to assess influences of MEG3/miR-494-3p/OTUD4 axis on cell viability, migration, and invasion. The results illustrated that overexpressed MEG3 decreased cell viability, migratory and invasive abilities while silencing OTUD4 in MEG3-overexpressed cells could partially alleviate the inhibition (Fig. 4C–E).

### Overexpressed MEG3 inhibits tumorigenic ability of breast cancer in vivo

**Fig. 5:**
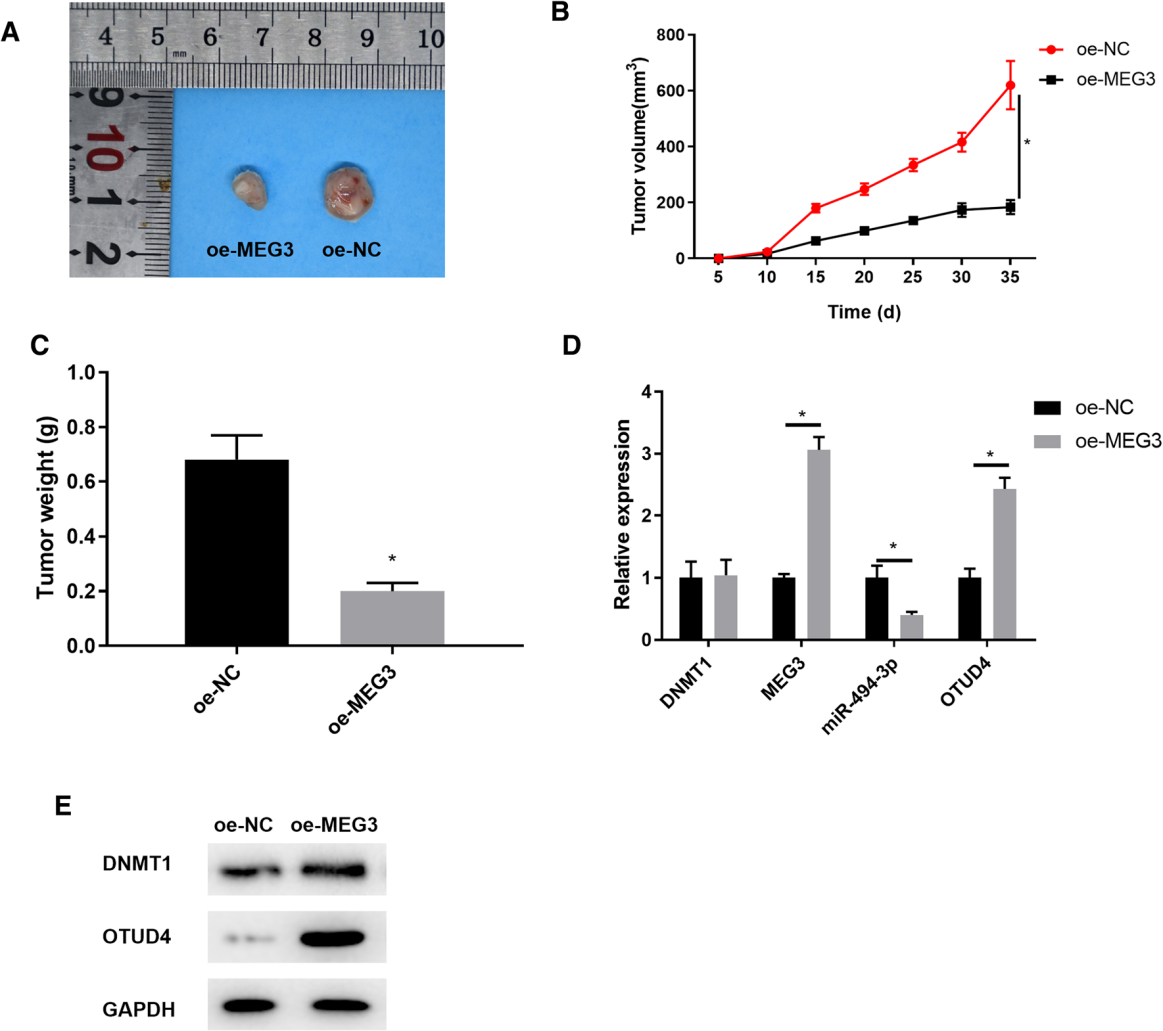
Upregulation of MEG3 inhibits breast cancer cell tumorigenesis in vivo. **A** tumor stereogram, **B** tumor volume and **C** tumor weight of nude mice in each group were measured; **D** DNMT1, MEG3, miR-494-3p and OTUD4 expression levels upon MEG3 overexpression were detected by qRT-PCR and **E** protein expression levels of DNMT1 and OTUD4 were tested by western blot. *p  < 0.05

Finally, to investigate whether MEG3 influences breast cancer cell growth in vivo, we overexpressed MEG3 in nude mice to observe effect of MEG3 on the tumorigenicity of breast cancer. The tumor weight and volume of each group were detected (Fig. 5A–C). Up-regulating MEG3 reduced tumor volume and weight. DNMT1, MEG3, miR-494-3p and OTUD4 expression upon MEG3 overexpression was detected by qRT-PCR (Fig. 5D), while protein expression of DNMT1 and OTUD4 was tested by western blot (Fig. 5E). Results exhibited that miR-494-3p was remarkably down-regulated when MEG3 was upregulated, while OTUD4 were remarkably up-regulated (*p*  < 0.05). Besides, overexpression of MEG3 did not affect DNMT1 expression significantly (*p*  > 0.05).

## Discussion

Expression of abnormal DNA methylated lncRNAs is a key epigenetic mechanism associated with cancer progression [30]. Previous studies demonstrated that DNMT1 (DNA methylase) can promote the methylation of lncRNA MEG3, and methylation of MEG3 promoter along with changes in gene region is a primary reason for MEG3 aberrant expression in tumors [31]. Gao et al. [32] found that DNMT1 protein promotes the proliferation of retinoblastoma by silencing MEG3. Wang et al. [16] revealed that miR-506/SP3/SP1/DNMT1/MEG3s is a new regulatory axis for migration and invasion of breast cancer cell lines. Our experimental results are consistent with the findings of Wang et al. The detection showed high DNMT1 expression and low MEG3 expression. Silencing DNMT1 reduced MEG3 promoter methylation level and upregulated MEG3 expression. We also found that down-regulation of SNMT1 hampered progression of breast cancer cells, which could be reversed by MEG3 silencing. Additionally, up-regulating MEG3 inhibited growth of breast cancer.

MEG3 is located in the DK1-MEG3 imprinting region on chromosome 14, and the region contains multiple imprinted genes and some miRNAs [33]. This feature makes MEG3 a key lncRNA that modulates different types of genes. MEG3 can function as a ceRNA in tumors [21]. For example, lncRNA MEG3 is less expressed in prostate cancer, which affects cell proliferation, migration, invasion ability and apoptosis rate by regulating miR-9-5p and QKI-5 [34]. MEG3 may exert anti-tumor function in pathogenesis of colorectal cancer by regulating miR-376/PRDK1 signal axis, and MEG3 may become a new target for colorectal cancer treatment [35]. Despite this, MEG3 is less studied, especially in breast cancer. Herein, we proved through RIP and dual luciferase experiment that MEG3 acted as a ceRNA of miR-494-3p in breast cancer. MEG3 carries the binding sequence of miR-494-3p and adsorbs miRNA like a sponge. As a result, miR-494-3p was bound to MEG3, resulting in the decreased expression level of miR-494-3p. Moreover, the binding of miR-494-3p with its target mRNA was prevented to some extent. In vitro functional experiments verified that upregulation of miR-494-3p could restore inhibitory effect of MEG3 overexpression on malignant behavior of breast cancer cells.

Numerous evidence suggests that lncRNAs are involved in regulating their downstream genes as ceRNAs [36]. OTUD4 encodes for a protein of 495 amino acids with an OTU domain and plays a role via their enzymatic deubiquitinating activities [37]. Zhao et al. [38] discovered that OTUD4, which is downregulated in 11 human cancers, may be a biomarker for prognosis prediction in a variety of cancers. OTUD4 has been found to be lowly expressed in non-small cell lung cancer and is able to inhibit proliferation of cancer cells [39]. In addition, alkylation damage which is critical for cancer chemotherapy can also be regulated by OTUD4 [40]. However, the mechanism of OTUD4 in breast cancer has not been reported. We revealed for the first time that MEG3 regulated OTUD4 in breast cancer cells through competitively binding of miR-494-3p, thus regulating growth of breast cancer cells. Besides, Patrick William Jaynes et al. [38] found that OTUD4 is associated with TGFβ signaling transduction in cancer, and OTUD4 regulates TGFβ pathway in breast cancer. Combined with the above literature, we speculated that OTUD4 in this study might regulate progression of cancer cells by mediating TGFβ pathway.

## Conclusions

**Fig. 6:**
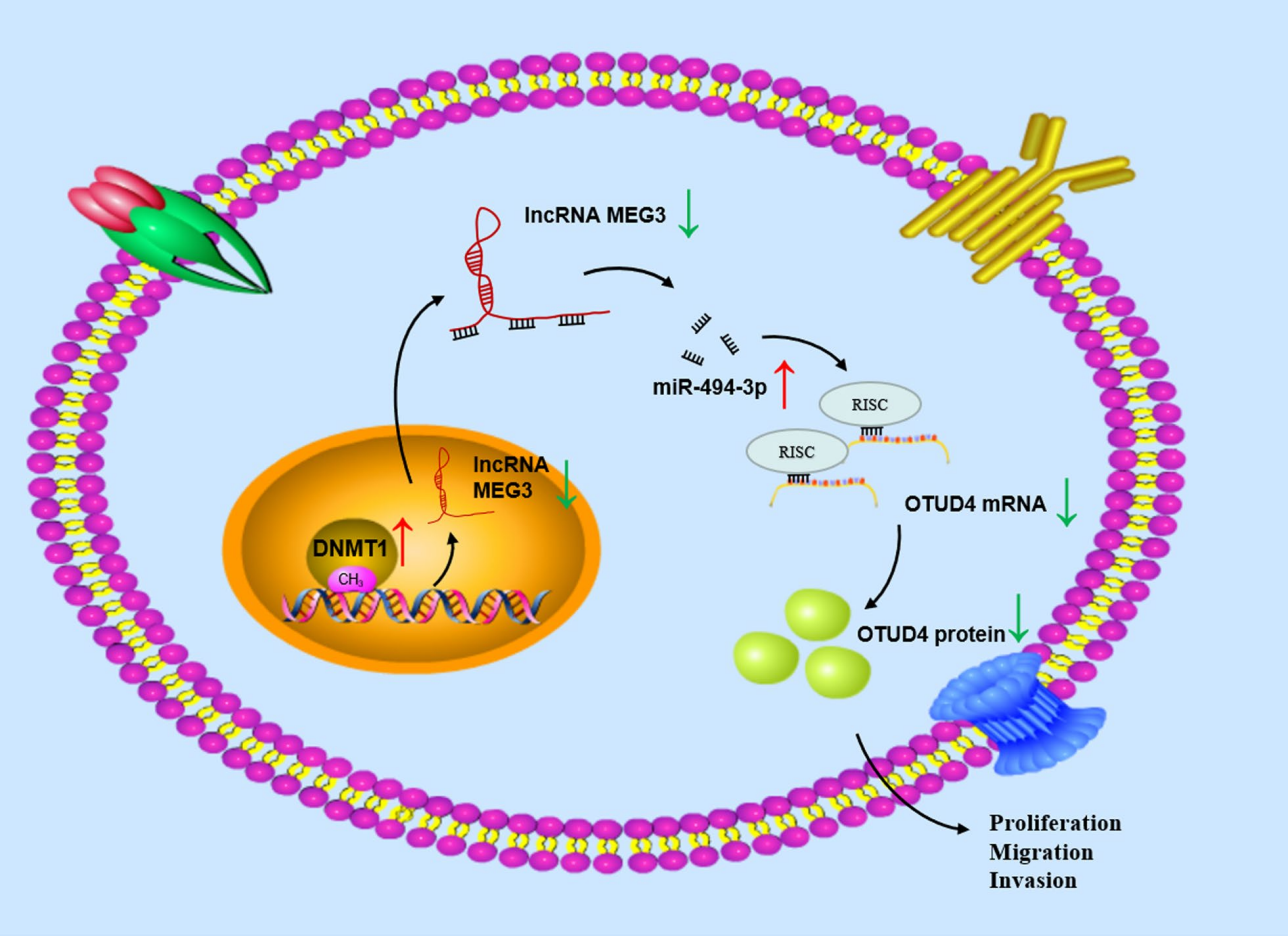
The molecular mechanism of the DNMT1/MEG3/miR-494-3p/OTUD4 axis in breast cancer

Anyway, our study first found DNMT1/MEG3/miR-494-3p/OTUD4 axis (Fig. 6). We also proved that MEG3 overexpression effectively inhibited development of breast cancer cells by targeting miR-494-3p/OTUD4 axis. Upregulation of MEG3 suppressed the growth of breast cancer. Although experimental design of this study is reasonable and evidence is substantial, the results have not been verified at clinical level. Nevertheless, new mechanisms for breast cancer progression and potential therapeutic targets were revealed here. In the future, we will further verify application value of MEG3 as a therapeutic target in clinical practice. Correlation between MEG3 and the prognosis and clinicopathology of breast cancer patients will be further discussed. Possible value of MEG3 as a prognostic factor or diagnostic marker for patients will be evaluated.

## Supplementary Information

**Additional file 1: Figure S1.** Flow chart of the study on DNMTI/MEG3/miR-494-3p/OTUD4 axis affecting progression of breast cancer

## Data Availability

The data and materials in the current study are available from the corresponding author on reasonable request.
