# Supplementary figures and images for: DNMT1 facilitates growth of breast cancer by inducing MEG3 hyper-methylation
Source: Cancer Cell Int. 2022 Feb 2;22:56. doi: 10.1186/s12935-022-02463-8 (PMC8812010; doi:10.1186/s12935-022-02463-8)

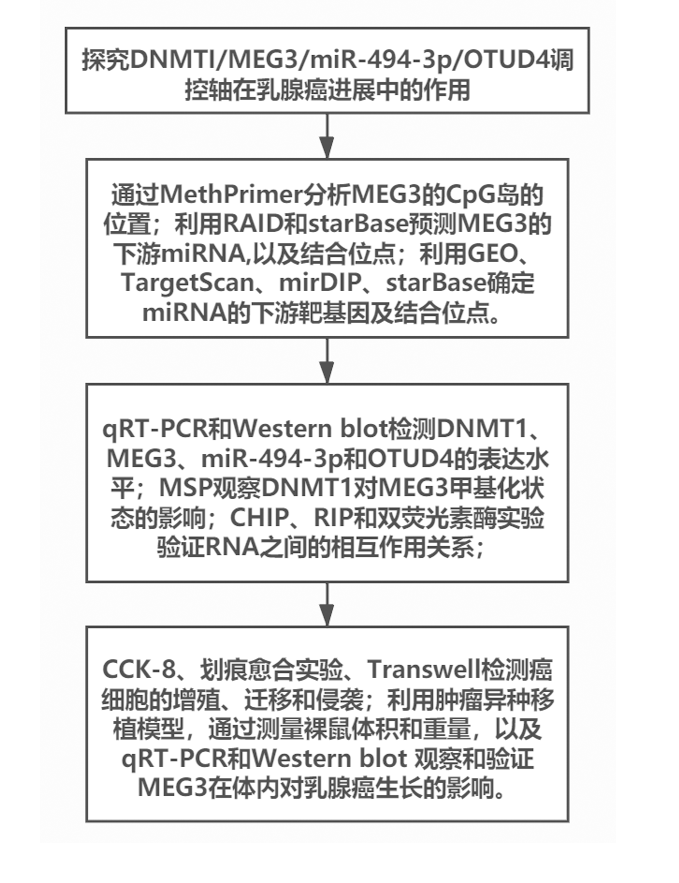

Supplement: Supplementary file 1 — Additional file 1: Figure S1. Flow chart of the study on DNMTI/MEG3/miR-494-3p/OTUD4 axis affecting progression of breast cancer [file 12935_2022_2463_MOESM1_ESM.tif]
